# Sleep monitoring based on triboelectric nanogenerator: wearable and washable approach

**DOI:** 10.3389/fpsyt.2023.1163003

**Published:** 2023-05-16

**Authors:** Zhiyuan Zhu, Maoqiu Pu, Zisheng Xu

**Affiliations:** ^1^College of Electronic and Information Engineering, Southwest University, Chongqing, China; ^2^Key Laboratory of the Ministry of Education for Advanced Catalysis Materials, Zhejiang Normal University, Jinhua, China

**Keywords:** triboelectric nanogenerator, sleep monitoring, portable equipment, smart mattress, smart pillow

## Introduction

In order to further promote miniaturization, convenience and intelligence of sleep monitoring devices, a novel method using triboelectric nanogenerator (TENG) for sleep monitoring and analysis has attracted attention gradually (1–9). TENG is an energy collection technology (10–12), which based on coupling of triboelectric electrification and electrostatic induction during the conversion of mechanical energy to electrical energy (13). with advantages of low cost, diverse structure, stable output, high energy conversion efficiency, strong shape adaptability, eco-friendly (14, 15), TENG can harvest mechanical energy from water waves, vibration, raindrops, wind and other environments (16). Its unique working modes also enables TENG to obtain biomechanical energy under sports and physiological conditions (17), including body movement, respiration, and heartbeat, which enables sleep monitoring free from the lack of batteries with a smaller volume and better use experience.

**Figure 1:**
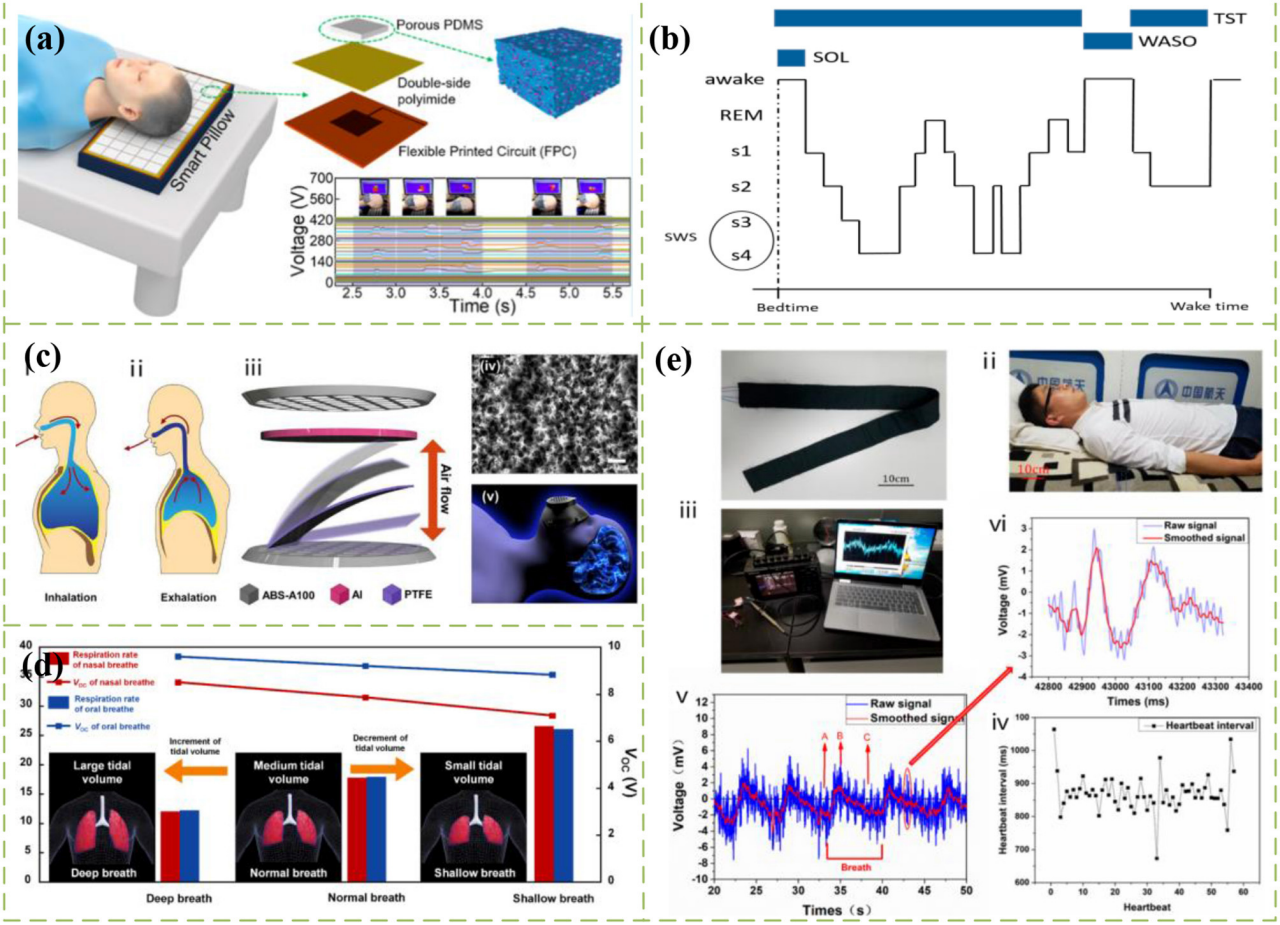
**(a)** Self-powered intelligent pillow based on TENG monitors head movement track (9). **(b)** An example of hypnogram (21). **(c)** Self-powered mask shape wearable device based on TENG (28). **(d)** Schematic diagram of breathing mode (28). **(e)** Test process of sleep monitoring belt made by porous TENG self-powered tactile sensor with CNT doping (3).

At present, sleep monitoring based on TENG mainly focuses on respiratory rate, head movement, eye changes and limb movements, which are important parameters related to sleep quality. For example, Yue et al. (1) prepared a self-powered all-nanofiber electronic skin based on TENG, which integrates a series of complex flexible sensors to analyze sleep quality by detecting respiratory rate and carotid signal frequency. However, the preparation process is relatively complex. Zhang et al. (5) fabricate a self-powered waist wearable respiratory monitoring device by taking advantage of the characteristics that the abdomen will deform during breathing. The respiratory information is retrieved by detecting the changes in abdominal circumference during human respiration to monitor sleep status. In addition, the energy generated by clearing abdominal deformation during breathing can also provide energy for the sensor. Kou et al. (9) prepared an intelligent pillow based on a flexible and breathable triboelectric nanogenerator (FB-TENG) sensor array, and evaluated the sleep quality by detecting the real-time head movement trajectory during sleep by the self-powered pressure sensor array, as shown in Figure 1a. On the other hand, Cao et al. (18) proposed a free deformable tribo-sensor (FDTS) based on nanofiber reinforced ultra-thin elastomer. After integrating the FDTS with the eye mask, the sleep state is evaluated by sensing the blinking action of the human body during sleep. In the research of limb motion, there are many sensitive and TENG-based limb motion sensors (7, 8). By monitoring the voltage output signal generated by the relative motion of the triboelectric layer in the device caused by human body movement in sleep, the time and number of human body movements in sleep can be collected and recorded in real time. The monitoring of sleep quality is mainly realized by pressure sensors, but the existing monitoring system still needs to improve the

sensitivity and stability of detection and faces challenges such as complex structure, high manufacturing cost, and difficulty in washing, which limits the application in real-time sleep posture detection and physiological analysis.

This paper analyzes the work of current researchers in respiratory rate, head movement, eye changes and limb movements, which are important parameters related to sleep quality. At the same time, it also analyzes the development direction of emerging electronic devices for sleep monitoring and analysis in the future, which has certain reference significance.

## TENG and sleep disorders

Due to the accelerated pace of modern life, more and more people are experiencing sleep disorders such as insomnia, obstructive sleep apnea-hypopnea syndrome (OSAHS), and circadian rhythm disorders, often caused by increased work pressure and irregular lifestyles (19, 20). Effective sleep monitoring and analysis are crucial in identifying sleep quality problems promptly, adjusting health habits, and seeking medical advice in a timely manner (21, 22). Figure 1b displays a typical sleep architecture, which represents the cyclic pattern of sleep (21). Numerous energy harvesters were developed in the past decades (23). Wherein TENGs can convert mechanical energy in the environment into electrical energy for self-power supply, and obtain information regarding environmental changes by analyzing the generated electrical signals (24, 25), even improve the overall performance of the system (26). In addition, these sleep monitoring devices generally use a sensitive motion sensor based on TENG. The relative movement of the triboelectric layer in the device which is caused by the tiny movements of the human body during sleep can generate a voltage output signal of up to tens of volts, thus reflecting the sleep situation in real time. For instance, we can analyze the signal frequency to determine wakefulness, shallow sleep, and deep sleep stages, as well as use the recorded time to determine the sleep duration and time taken to fall asleep.

## High sensitive triboelectric nanogenerator wearable devices

To facilitate and quickly implement sleep monitoring, TENGs are usually fabricated as various wearable devices, such as electronic skin patches (7), belts (5), goggles (18), which can analyze sleep behavior by monitoring limb movements, breathing or eye movements. In recent years, there has been a lot of research on the performance improvement and function optimization of wearable devices for TENG. For example, Chen et al. (27) vertically integrated the single-electrode TENG and the fiber-based piezoelectric nanogenerators (PENG) to further improve the self-power efficiency of wearable devices. At the same time, the piezoelectric nanofibers are used to monitor human physiological signals quantitatively and realize sleep monitoring. Salauddin et al. (6) combined MXene/Ecoflex nanocomposites with fabric to build a waterproof triboelectric nanogenerator device (FW-TENG) with advantages of self-powering and mechanical comfortable. Besides, it is also very reliable and stable underwater, which is an important feature for next generation of wearable/portable technology. On the optimization of data processing based on TENG wearable devices, Yun et al. (28) proposed a mask-shaped triboelectric nanogenerator (M-TENG) using mechanical energy generated by respiratory airflow, as shown in Figure 1c. This wearable device can detect the electrical signals generated by the breathing patterns in different sleep stages (as shown in Figure 1d). These electrical signals can be classified with the assistance of machine learning. Considering the optimization of sensor preparation methods for wearable devices, Ding et al. (3) designed a self-powered tactile sensor with CNT-doped porous TENG. This self-powered sensor has low cost, high durability and good sensitivity. In the test, the sleep monitoring belt prepared by this sensor can obtain accurate data about real-time heartbeat and respiration, as shown in Figure 1e.

Although wearable devices based on TENGs have achieved great progress in recent years, it is inevitable that wearable devices will affect the natural sleep state of the human body to some extent during use. In this regard, many researchers start with intelligent textiles based on TENG, and prepare them into more comfortable bedding to maximize the restoration of the natural sleep state of the human body.

## Washable intelligent mattress for sleep monitoring

A non-invasive sleep monitoring scheme that does not need to attach any sensor or transducer to the human body has been proposed. The method is to use large pressure-sensitive and washable smart textiles based on TENG array to make them into smart mattresses, which can detect sleep behavior in real time. This smart mattress is mainly composed of three layers: the top layer and the bottom layer are composed of cross and vertical conductive layers, and the middle of the two conductive layers is a wavy polyethylene terephthalate (PET) film interlayer. When external pressure is applied to the smart mattress, the structural change of the middle PET layer will lead to the change of its contact area with the two conductive layers, thus producing potential difference. In current work, the intelligent mattress which proposed by Lin et al. (4) has excellent pressure sensitivity (0.77 V/Pa), fast response time (80 ms), and can still generate stable external pressure signal after washing in tap water. However, the wave-shaped structure embedded in its mattress lacks comfortable materials, which can bring a certain sense of discomfort. Moreover, the size magnitude of the array element is 10 × 10 cm^2^, resulting in low resolution. Further, Zhou et al. (2) proposed a single layer soft intelligent textile that can be used for mattresses has solved the issue of comfort in terms of material selection, but its resolution remains the same. Intelligent mattress devices generally offer the advantages of low cost, fast response time, and deep stability. However, their sleep monitoring detection methods are relatively simple, which makes it difficult to accurately reflect specific sleep statuses.

## Head movement monitoring during sleep

Considering the relationship between head posture changes and sleep state during sleep, an intelligent pillow which based on a flexible and breathable triboelectric nanogenerator (FB-TENG) sensor array was developed (9). This method uses intelligent pillow to realize sleep state monitoring with characteristics of high resolution, pressure sensitivity, non-intrusive, comfort and breathability. Each FB-TENG sensor unit that touched by the human head will output a voltage signal during sleep, and the output voltage signal which is generated by each FB-TENG sensor unit is different due to the different touching force. It can monitor and record the movement track of the human head by sorting each voltage output signal in chronological order. These changes are mainly caused by body turnover, and then these can be used to analyze the change of sleeping posture and reflect the sleep situation directly. In addition, the intelligent pillow does not need to wear any wearable electronic equipment during use, which can greatly reduce the impact of monitoring equipment on the human body's natural sleep state.

## Discussion

Through the coupling effect of triboelectric electrification and electrostatic induction, TENG is not only a low-cost and reliable energy collection technology, but also a combination of self-driving pressure sensing technology to achieve the monitoring of sleep quality. The only restriction on its application is the anti-cleaning performance of materials. Some of the sleep monitors need to be worn by users, which can't maintain the most natural state of sleep. And the use of patches or electronic skin with poor permeability may also cause skin itching, inflammation and other uncomfortable symptoms. In this regard, a large pressure-sensitive and washable smart textile which based on the TENG array can be used to make intelligent instruments, it can detect sleep behavior in real time, such as smart mattresses, smart pillows, etc. Among them, the TENG unit is made of conductive material and elastomer material of waveguide structure, with excellent characteristics such as high sensitivity, fast response time, durability and water resistance, and is connected to form a self-driving pressure sensor array. At the same time, it is equipped with an additional integrated data acquisition, processing and wireless transmission system, which can realize real-time sleep behavior monitoring and sleep quality evaluation. Therefore, the application of nano-triboelectric generator in the field of bedding is more feasible and available than the solution of using wearable devices.

On the other hand, to further improve the accuracy and specificity of sleep monitoring, an interesting idea is to use TENG based smart textiles with smart pillows in conjunction, and comprehensive analysis of sleep data with machine learning which is obtained from multiple intelligent bedding products, so as to provide targeted recommendations for different sleep behaviors of different patients. Further, because of the real-time collection of patients sleeping posture through intelligent bedding, it is possible to achieve poor sleeping posture reminders. For infants and incapacitated patients, smart textiles and smart pillows can add certain control programs to prevent falling out of bed issues. For example, an alarm function can be added to the TENG arrays in the row closest to the edge of the bed for smart pillows or smart textiles, and trigger the alarm function when they are contacted simultaneously.

Moreover, TENG can harvest mechanical energy from body organs (heart beating, muscle contraction and gastrointestinal peristalsis) to form electric field and generate electricity to realize electrical stimulation, which can help regulate the heartbeat, relieve muscle atrophy and promote wound healing. At the same time, the electricity which generated by TENG can be used to stimulate cells, tissues and organs directly (29). This shows the potential application in rehabilitation and treatment. Secondly, different external stimulation may lead to different output signals of TENG, which can work as self-powered sensors to monitor real-time physiological signals. In addition, in order to improve stability and provide more choices of triboelectric materials, TENG is integrated with other sensors to form an impedance matching system or hybrid system. TENG has great potential in the field of medical care, but it still has a certain distance between experiment and practical application.

## Ethics statement

Written informed consent was obtained for the publication of any potentially identifiable images or data included in this article.

## Author contributions

Writing—review and project administration: ZZ. Writing—original draft preparation and editing: MP. Investigation and supervision: ZX. All authors have read and agreed to the published version of the manuscript.

## References

[1] Yue O Wang X Hou M Zheng M Bai Z Cui B. Skin-inspired wearable self-powered electronic skin with tunable sensitivity for real-time monitoring of sleep quality. Nano Energy. (2022) 91:106682. 10.1016/j.nanoen.2021.106682

[2] Zhou Z Padgett S Cai Z Conta G Wu Y He Q. Single-layered ultra-soft washable smart textiles for all-around ballistocardiograph, respiration, and posture monitoring during sleep. Biosens Bioelectron. (2020) 155:112064. 10.1016/j.bios.2020.112064 32217330

[3] Ding X Cao H Zhang X Li M Liu Y. Large scale triboelectric nanogenerator and self-powered flexible sensor for human sleep monitoring. Sensors. (2018) 18:1713. 10.3390/s18061713 29799495 PMC6022135

[4] Lin Z Yang J Li X Wu Y Wei W Liu J. Large-scale and washable smart textiles based on triboelectric nanogenerator arrays for self-powered sleeping monitoring. Adv Funct Mater. (2018) 28:1704112. 10.1002/adfm.201704112

[5] Zhang H Zhang J Hu Z Quan L Shi L Chen J. Waist-wearable wireless respiration sensor based on triboelectric effect. Nano Energy. (2019) 59:75–83. 10.1016/j.nanoen.2019.01.063

[6] Salauddin M Rana SMS Sharifuzzaman M Rahman MT Park C Cho H. A novel MXene/Ecoflex nanocomposite-coated fabric as a highly negative and stable triboelectric layer for high-output triboelectric nanogenerators. Adv Energy Mater. (2021) 11:2002832. 10.1002/aenm.202002832

[7] Li R Wei X Xu J Chen J Li B Wu Z. Smart wearable sensors based on triboelectric nanogenerator for personal healthcare monitoring. Micromachines. (2021) 12:352. 10.3390/mi12040352 33806024 PMC8064435

[8] Song WX Gan B Jiang T Zhang Y Yu A Yuan H. Nanopillar arrayed triboelectric nanogenerator as a self-powered sensitive sensor for a sleep monitoring system. ACS Nano. (2016) 10:8097–103. 10.1021/acsnano.6b04344 27494273

[9] Kou H Wang H Cheng R Liao Y Shi X Luo J. Smart pillow based on flexible and breathable triboelectric nanogenerator arrays for head movement monitoring during sleep. ACS Appl Mater Interfaces. (2022) 14:23998–4007. 10.1021/acsami.2c03056 35574831

[10] Gao H Hu M Ding J Xia B Yuan G Sun H. Investigation of contact electrification between 2D MXenes and MoS2 through density functional theory and triboelectric probes. Adv Funct Mater. (2023) 2213410. 10.1002/adfm.202213410

[11] Wei X Wang B Wu Z Wang ZL. An open-environment tactile sensing system: toward simple and efficient material identification. Adv Mater. (2022) 34:2203073. 10.1002/adma.202203073 35578973

[12] Xu Z Bao K Di K Chen H Tan J Xie X. High-performance dielectric elastomer nanogenerator for efficient energy harvesting and sensing via alternative current method. Adv Sci. (2022) 9:2201098. 10.1002/advs.202201098 35396790 PMC9218771

[13] Zhang C Tang W Han C Fan F Wang ZL. Theoretical comparison, equivalent transformation, and conjunction operations of electromagnetic induction generator and triboelectric nanogenerator for harvesting mechanical energy. Adv Mater. (2014) 26:3580–91. 10.1002/adma.201400207 24677413

[14] Li W Torres D Díaz R Wang Z Wu C Wang C. Nanogenerator-based dual-functional and self-powered thin patch loudspeaker or microphone for flexible electronics. Nat Commun. (2017) 8:15310. 10.1038/ncomms15310 28508862 PMC5440853

[15] Wang J Wu C Dai Y Zhao Z Wang A Zhang T. Achieving ultrahigh triboelectric charge density for efficient energy harvesting. Nat Commun. (2017) 8:88. 10.1038/s41467-017-00131-4 28729530 PMC5519710

[16] Wang ZL. New wave power. Nature. (2017) 542:159–60. 10.1038/542159a 28179678

[17] Xu Q Fang Y Jing Q Hu N Lin K Pan Y. A portable triboelectric spirometer for wireless pulmonary function monitoring. Biosens Bioelectron. (2021) 187:113329. 10.1016/j.bios.2021.113329 34020223 PMC8118703

[18] Cao R Zhao S Li C. Free deformable nanofibers enhanced tribo-sensors for sleep and tremor monitoring. Acs Appl Electr Mater. (2019) 1:2301–7. 10.1021/acsaelm.9b00483

[19] King S Cuellar N. Obstructive sleep apnea as an independent stroke risk factor: a review of the evidence, stroke prevention guidelines, and implications for neuroscience nursing practice. J Neurosci Nurs. (2016) 48:133–42. 10.1097/JNN.0000000000000196 27136407

[20] Koehler U Cassel W Hildebrandt O Kesper K Kianinejad P Nell C. Obstructive sleep apnea in neurological diseases: specially as a risk factor for stroke. Nervenarzt. (2014) 85:35–42. 10.1007/s00115-013-3890-9 24362594

[21] Crivello A Barsocchi P Girolami M Palumbo F. The meaning of sleep quality: a survey of available technologies. IEEE access. (2019) 7:167374–90. 10.1109/ACCESS.2019.295383527409075

[22] Sigurdson K Ayas NT. The public health and safety consequences of sleep disorders. Can J Physiol Pharmacol. (2007) 85:179–83. 10.1139/y06-095 17487258

[23] Xu Z Tan J Chen H Di K Bao K Cheng J. Fatigue-resistant high-performance dielectric elastomer generator in alternating current method. Nano Energy. (2023) 109:108314. 10.1016/j.nanoen.2023.108314

[24] Wen Z Guo H Zi Y Yeh MH Wang X Deng J. Harvesting broad frequency band blue energy by a triboelectric-electromagnetic hybrid nanogenerator. ACS Nano. (2016) 10:6526–34. 10.1021/acsnano.6b03293 27267558

[25] Yun J Roh H Choi J Gu D Kim D. Disk Triboelectric nanogenerator-based nonvolatile memory toward smart identification system. Adv Funct Mater. (2021) 31:2102536. 10.1002/adfm.202102536

[26] Zhang J Xu Q Li H Zhang S Hong A Jiang Y. Self-powered electrodeposition system for sub-10-nm silver nanoparticles with high-efficiency antibacterial activity. J Phy Chem Lett. (2022) 13:6721–30. 10.1021/acs.jpclett.2c01737 35849530

[27] Chen X Song Y Su Z Chen H Cheng X Zhang J. Flexible fiber-based hybrid nanogenerator for biomechanical energy harvesting and physiological monitoring. Nano Energy. (2017) 38:43–50. 10.1016/j.nanoen.2017.05.047

[28] Yun J Park J Jeong S Hong D Kim D. A mask-shaped respiration sensor using triboelectricity and a machine learning approach toward smart sleep monitoring systems. Polymers. (2022) 14:3549. 10.3390/polym14173549 36080623 PMC9460850

[29] Wei X Wang Y Tan B Zhang E Wang B Su H. Triboelectric nanogenerators stimulated electroacupuncture (EA) treatment for promoting the functional recovery after spinal cord injury. Materials Today. (2022) 60:41–51. 10.1016/j.mattod.2022.09.010

